# Cross-sectional association of light sensor-measured time outdoors with physical activity and gross motor competency among U.S. preschool-aged children: the 2012 NHANES National Youth Fitness Survey

**DOI:** 10.1186/s12889-022-13239-0

**Published:** 2022-04-26

**Authors:** Soyang Kwon, Pooja S. Tandon, Meghan E. O’Neill, Adam B. Becker

**Affiliations:** 1 Ann & Robert H. Lurie Children’s Hospital of Chicago, 225 E Chicago Ave. Box 157, Chicago, IL 60611 USA; 2 University of Washington & Seattle Children’s Hospital, PO Box 5371, M/S CURE-3, Seattle, WA 98145 USA

**Keywords:** Preschoolers, ActiGraph, Monitor-independent movement summary (MIMS) outside, Outdoor play, Gross motor skills

## Abstract

**Background:**

Time spent outdoors (outdoor time) has been suggested to be beneficial for physical activity (PA) and healthy development among preschool-aged children. The aim of this study was to quantify PA level and gross motor competency associated with light sensor-measured daily outdoor time in a representative sample of U.S. children aged 3 to 5 years.

**Methods:**

The study sample included 301 participants (149 girls) aged 3 to 5 years from the 2012 U.S. National Health and Examination Survey National Youth Fitness Survey. ActiGraph GT3X+ accelerometers with a built-in ambient light sensor were used to measure PA (expressed in monitor-independent movement summary [MIMS]) and outdoor time. The Test of Gross Motor Development-Second Edition (TGMD-2) was used to assess gross motor skills. Multivariable linear regression models were fit to predict daily and gross motor scores by daily outdoor time.

**Results:**

Average daily outdoor time was 95 min (median of 84 min; interquartile range of 52 to 123 min). Means of daily outdoor time and daily MIMS were not significantly different between boys and girls. Among girls, every additional 10 min of daily outdoor time was associated with an additional 540 daily total MIMS (95% CI = 372, 708). Among boys, every additional 10 min of daily outdoor time was associated with an additional 296 daily total MIMS (95% CI = 131, 460). Every additional 10 min of daily outdoor time was associated with a 0.1-point (95% CI = 0.001, 0.130) higher object control standard score. Daily outdoor time was not associated with a locomotor standard score.

**Conclusions:**

In a representative sample of U.S. preschool-aged children, daily outdoor time was positively associated with daily PA. The contribution of outdoor time to PA was greater among girls than boys, suggesting that providing outdoor opportunities is critical for promoting PA, particularly among girls.

**Supplementary Information:**

The online version contains supplementary material available at 10.1186/s12889-022-13239-0.

## Background

The preschool years are a critical period for establishing physical activity (PA) habits [1] and developing fundamental motor skills such as running, jumping, throwing, and catching [2]. Some studies [3, 4], though not of representative U.S. samples, have shown that a substantial proportion of U.S. preschool-aged children (3 to 5 years of age) do not engage in recommended levels of PA. In particular, preschool-aged girls were reported to be less active than preschool-aged boys [3–7].

Spending time outdoors (outdoor time) has been suggested to be beneficial for PA and healthy development among children [8, 9], including preschool-aged children [6]. Although study results are mixed [10, 11], a positive impact of outdoor time on gross motor development among preschool-aged children has also been suggested. However, outdoor time has decreased over the years among children globally [8, 12]. Furthermore, one study [13] showed that preschool-aged children in the U.S. spent much less time outdoors at preschool compared to those in Sweden (18% vs. 47% of approximately 8 h of preschool attendance). However, a knowledge gap exists regarding national estimates of outdoor time and quantification of the association between outdoor time and PA in U.S. preschool-aged children.

To accurately quantify the contribution of outdoor time to PA in a free-living setting, an accurate assessment of outdoor time is required. While direct observation of outdoor time is a valid method [14], it is not feasible to employ 24- h a day over multiple days in a large-scale epidemiologic study. A proxy-report has been used to estimate daily outdoor time [9–11, 15], but it is prone to measurement error. A few studies [16–20] have used sensors, such as Global Positioning Systems (GPS). For example, Tandon et al. [19] reported that every additional 10 min of GPS-measured outdoor time was associated with additional 2.9-min of accelerometer-measured moderate- and vigorous-intensity PA at childcare settings (2.7 min for girls and 3.0 min for boys) among 46 children. However, use of GPS devices has limitations such as satellite signal losses and inaccurate GPS positions in some settings [16], in addition to the participant burden of wearing both an accelerometer and a GPS device. The ActiGraph GT3X+ accelerometer includes a built-in ambient light sensor. Flynn et al. [21] demonstrated that light sensor-measured light intensity, expressed in lux, can be used to accurately distinguish between indoor and outdoor conditions among preschool-aged children in a free-living environment.

In 2012, the U.S. National Health and Examination Survey (NHANES) National Youth Fitness Survey (NNYFS) was conducted as a NHANES ancillary study to evaluate PA, fitness (3 to 15 years of age), and gross motor development (3 to 5 years of age) in a representative sample of U.S. youth. Utilizing the NNYFS data, the primary aim of this study was to quantify time spent in PA associated with outdoor time in a representative sample of U.S. children aged 3 to 5 years. The secondary aim was to examine the association between outdoor time and gross motor competency.

## Methods

All methods were carried out in accordance with relevant guidelines and regulations.

### Participants

We conducted a secondary analysis using datasets from the 2012 NNYFS. Applying a complex staged sampling method and stratifying based on age and sex, NNYFS selected a representative sample of 368 U.S. children aged 3–5 years. Participants who were missing arms bilaterally were excluded from the NNYFS physical activity monitor (PAM) component. Participants who had physical or cognitive limitations described in the NNYFS protocol [22] were excluded from the NNYFS gross motor component. After exclusions, 352 participants were eligible for PAM and gross motor assessment components.

### Measurements

#### Accelerometer assessment

For the PAM component, ActiGraph model GT3X+ accelerometers (Pensacola, FL) were used to measure acceleration of body movement and ambient light levels. An accelerometer was programmed to record the magnitude of acceleration at 80 Hz sampling intervals (every 1/80th of a second) and ambient light at 1 Hz (every second), starting at the end of the participant’s examination visit (the 1st calendar day) and ending 8 days later (the 9th calendar day). The accelerometer was placed on a mesh wristband for use.

During the examination visit, participants were fitted with an accelerometer wristband (dorsal orientation) on the non-dominant hand. Participants were asked to keep wearing the wristband for 24 h/7 days (the 2nd to 8th calendar days) and remove the wristband on the morning of the 9th day. If participants needed to remove the accelerometer for any reason, they were instructed to put it back on the same wrist in the same orientation, as soon as possible. The accelerometer was returned by mail in postage-paid padded envelopes.

#### Accelerometer data processing

The publicly available PAM datasets contained acceleration data of the x-, y-, and z-axes expressed in Monitor-Independent Movement Summary (MIMS) units for each minute. The MIMS unit is a non-proprietary, open-source, device-independent universal summary metric [23]. The datasets also contained a variable to predict each minute as “wake” wear, “sleep” wear, “non-wear,” or “unknown,” based on a machine learning algorithm. The detailed process for the prediction can be found in the Analytic Notes of the NNYFS PAM data document [22].

Of the 352 eligible participants, 315 participants had at least 1 min of accelerometer data during the 7-day data collection period. We excluded minutes between 10:00 PM and 5:59 AM (treating the period as night sleep) [24]. We also excluded “non-wear” and “unknown” minutes to select wear (“wake” and “sleep”) minutes only. Finally, excluding any wear day with < 600 wear minutes, a total of 1829 days from 301 participants were selected as valid wear days. Participants with at least one valid wear day were included.

To estimate outdoor time, outdoor/indoor location was determined based on the lux values: ≥240 lx for outdoor and < 240 lx for indoor [21]. The threshold of 240 lx was shown to have 97% accuracy to classify indoor and outdoor conditions in a free-living environment [21]. For each valid wear day, outdoor time per day was calculated by summing outdoor minutes throughout the day. Daily outdoor time per person (minutes/day) was calculated by averaging daily outdoor minutes for valid wear days and was used as the primary exposure variable. Hourly outdoor time (minutes/hour) was calculated, only including hourly data with wear time ≥ 30 min.

To estimate PA levels, we additionally excluded 504 min (0.03%) in which MIMS could not be computed or data quality was flagged. Using MIMS triaxial values (the sum of MIMS x-, y-, and z-axis values; ranging from 0 to 283) [23], total MIMS per day was calculated by summing MIMS triaxial values throughout the day. Daily total MIMS (MIMS/day) per person were calculated by averaging total MIMS for valid wear days and were used as the primary outcome variables. Hourly total MIMS (MIMS/hour) was calculated, only including hourly data with wear time ≥ 30 min.

#### Gross motor assessment

Gross motor skills were evaluated using the Test of Gross Motor Development-Second Edition (TGMD-2). The TGMD-2 is a validated norm-referenced measure of common gross motor skills that develop early in life [22]. The TGMD-2 is composed of two gross motor subtests: locomotor and object control tests. The locomotor test measures the following six skills: run, gallop, hop, leap, horizontal jump, and slide. The object control test measures the following six skills: striking a stationary ball, dribble, kick, catch, overhand throw, and underhand roll. The detailed procedure manual for the TGMD-2 component is available at the NNYFS website [22]. A locomotor skill standard score (range of 1 to 20) and an object control skills standard score (range of 1 to 20) were calculated. The two standard scores were summed and converted into a gross motor quotient (range of 46 to 160) [25].

### Confounding variables

Based on our prior analyses [26], we considered several socioeconomic and familial characteristics, such as race/ethnicity, family income, presence of siblings in the household, and language spoken at home as potential confounding factors. During NNYFS in-person interviews, questions about socioeconomic and family characteristics were asked of an adult family member as a proxy for the participants, using the Computer-Assisted Personal Interviewing system. Race/ethnicity was categorized as Hispanic, non-Hispanic Black, non-Hispanic White, and other. Ratio of family income to poverty (family size-adjusted income level) was calculated as family income divided by poverty threshold. The ratio of family income to poverty was categorized as 0 to < 1.0 (below the poverty line), 1.0 to < 3.0, and ≥ 3.0 [26]. For the presence of siblings, we created two variables: (1) presence of sibling(s) ≤5 years old (yes or no), using responses to the question asking about the number of child(ren) ≤5 years old in the household and (2) presence of sibling(s) 6–17 years old (yes or no), using responses to the question asking about the number of child(ren) aged 6–17 years old in the household. Language spoken at home was categorized into either English only or at least some non-English.

### Statistical analysis

All analyses were conducted incorporating the complex sample design, such as weighting and clustering, using SAS 9.4 (Cary, NC). Missing data for gross motor variables (*n* = 10) were treated with a listwise deletion method. Missing data for family income (*n* = 14) were imputed as the middle category (1.0 to < 3.0). Descriptive analyses, including means and 95% confidence intervals (CIs), were conducted for the exposure and outcome variables.

To estimate daily total MIMS associated with daily outdoor time, we conducted multivariable linear regression analysis to predict daily total MIMS by daily outdoor time. The model was adjusted for the confounding variables that were statistically significantly (*p* < 0.05) associated with daily total MIMS in chi-square tests: age (3 [reference], 4, and 5 years), race/ethnicity (non-Hispanic White [reference], Hispanic, non-Hispanic Black, and other), ratio of family income to poverty (0 to < 1.0, 1.0 to < 3.0, or ≥ 3.0 [reference]), presence of sibling(s) ≤5 years old in the household (yes or no), and presence of sibling(s) 6–17 years old in the household. We also tested whether sex was a modifier using an interaction term. Sex-specific analyses were conducted only if sex was found to be a modifier.

To examine the association between daily outdoor time and gross motor competency, multivariable linear regression models were fit to predict the gross motor quotient, the locomotor standard score, and the object control standard score by daily outdoor time. The models were adjusted for the confounding variables that were statistically significantly (*p* < 0.05) associated with gross motor competency in chi-square tests: race/ethnicity, ratio of family income to poverty, and presence of sibling(s) 6–17 years old in the household. These models were not adjusted for sex or age, because the standard scores were sex- and age-adjusted scores. We also explored the association between PA and the gross motor scores using multivariable linear regression models.

## Results

Of the 352 eligible participants, 301 (149 girls; 49.5%) had at least one valid wear day. Compared to the 301 included participants, the 51 excluded participants tended to be younger (*p* = 0.06). Otherwise, the distributions of sex, race/ethnicity, and ratio of family income to poverty were similar. Of the 301 participants, 31.0% were 3 years old, 36.2% were 4 years old, and 32.8% were 5 years old. The racial/ethnic distribution of the sample was 54.0% non-Hispanic White, 25.1% Hispanic, 14.8% non-Hispanic Black, and 6.2% other.

Average daily “wake” wear time between 6 AM and 10 PM was 740 min (median: 786 min; interquartile range [IQR]: 730 to 824 min). The average number of valid wear days was 6 days (median: 6 days; IQR: 5 to 7 days). Only 9% of the sample had < 3 valid wear days. Average daily outdoor time was 95 min (median: 84 min; IQR: 52 to 123 min; 113 min for weekdays and 91 min for weekend days). Average daily total MIMS was 17,613 (median: 18,219 MIMS; IQR: 16,144 to 20,501 MIMS). Outdoor time and PA levels were not statistically different between boys and girls (Table 1).

**Table 1:** Means of accelerometer and gross motor measures in 2012 NNYFS participants aged 3 to 5 years

	Outdoor minutes/day	Total MIMS/day	Locomotor standard score^a^	Object control standard score^a^
	Mean ± SE	Mean ± SE	Mean ± SE	Mean ± SE
**All**	95 ± 8	17,613 ± 427	10.0 ± 0.2	8.6 ± 0.1
**Sex**				
Boy (*n* = 152)	97 ± 10	17,784 ± 595	9.4 ± 0.3	8.6 ± 0.2
Girl (*n* = 149)	93 ± 8	17,438 ± 432	10.7 ± 0.2	8.5 ± 0.2
**Age**				
3 years (*n* = 96)	83 ± 9	16,112 ± 727	9.4 ± 0.4	8.6 ± 0.3
4 years (*n* = 101)	101 ± 9	17,948 ± 579	10.5 ± 0.2	8.4 ± 0.3
5 years (*n* = 104)	100 ± 11	18,664 ± 287	10.1 ± 0.3	8.8 ± 0.3
**Race/ethnicity**				
Non-Hispanic White (*n* = 121)	102 ± 10	17,390 ± 616	9.6 ± 0.3	8.4 ± 0.2
Hispanic (*n* = 102)	84 ± 9	18,007 ± 526	10.4 ± 0.3	8.8 ± 0.2
Non-Hispanic Black (*n* = 61)	91 ± 9	18,209 ± 590	11.2 ± 0.6	9.0 ± 0.2
Other (*n* = 17)	89 ± 22	16,526 ± 1002	9.3 ± 1.2	7.6 ± 0.9
**Language spoken at home**				
English only (*n* = 203)	99 ± 9	17,666 ± 484	10.1 ± 0.2	8.6 ± 0.2
At least some non-English (*n* = 98)	82 ± 9	17,452 ± 610	10.0 ± 0.3	8.3 ± 0.2
**Ratio of family income to poverty**				
< 1.0 (below the poverty line; *n* = 112)	89 ± 9	17,803 ± 574	10.1 ± 0.3	8.8 ± 0.2
1.0 to < 3.0 (*n* = 110)	96 ± 8	17,889 ± 526	10.4 ± 0.4	8.5 ± 0.2
≥ 3.0 (*n* = 79)	101 ± 13	17,024 ± 552	9.5 ± 0.3	8.5 ± 0.3
**Sibling(s) in household**				
Yes: ≤ 5 years old and 6–17 years old (n = 61)	89 ± 12	17,454 ± 875	10.2 ± 0.6	8.4 ± 0.3
Yes: ≤ 5 years old only (*n* = 80)	91 ± 7	17,903 ± 534	10.2 ± 0.2	8.2 ± 0.2
Yes: 6–17 years old only (*n* = 118)	102 ± 11	17,565 ± 422	10.1 ± 0.4	9.1 ± 0.3
No (*n* = 42)	95 ± 11	17,316 ± 614	9.3 ± 0.7	8.0 ± 0.4

*MIMS* monitor-independent movement summary, *SE* standard error

^a^Missing *n* = 10

**Fig. 1:**
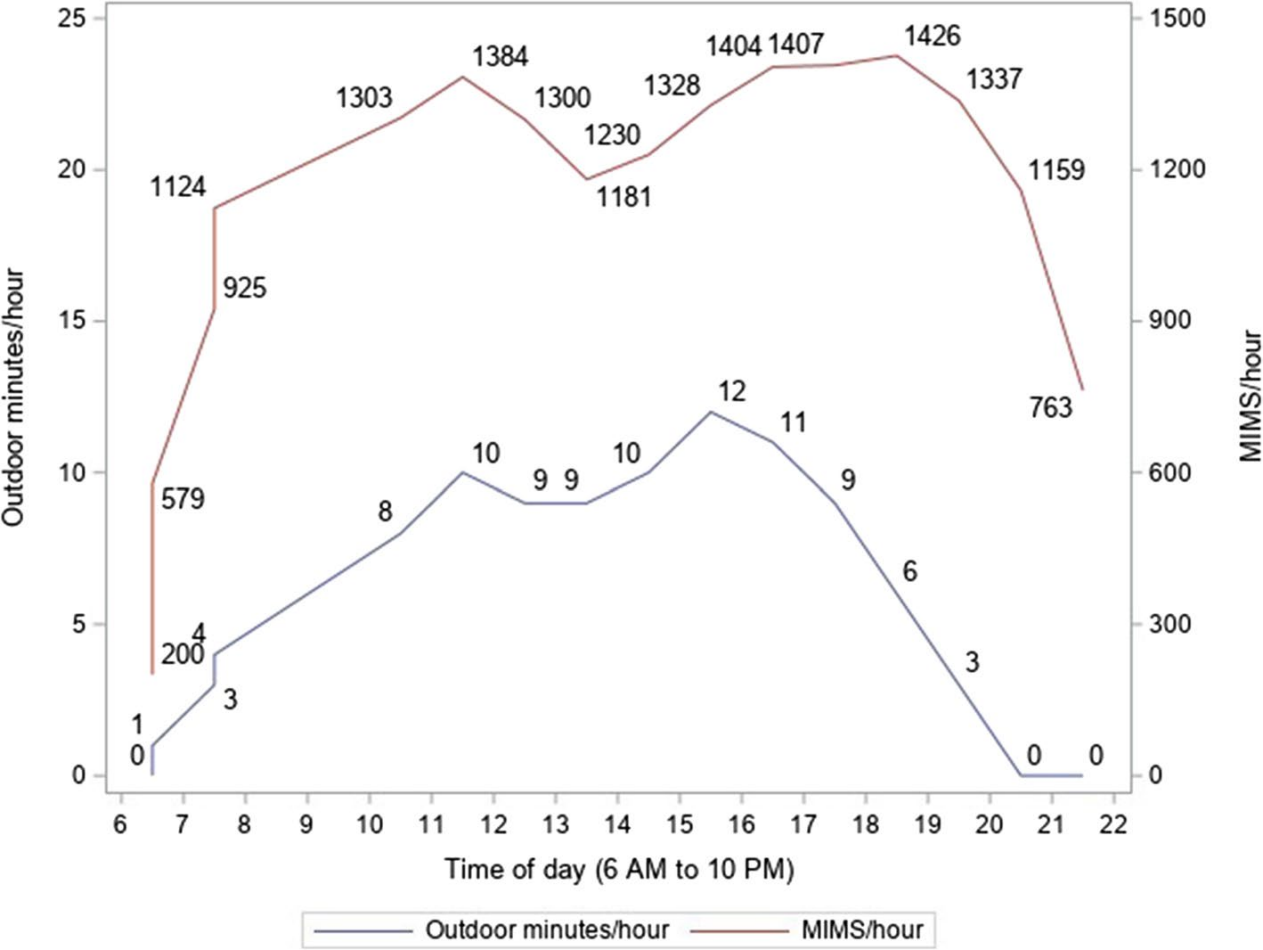
Hourly outdoor time and monitor-independent movement summary (MIMS) over the day in the 2012 NNYFS participants aged 3 to 5 years

Figure 1 illustrates hourly outdoor time and hourly MIMS between 6 AM and 10 PM. Hourly MIMS tended to be higher when hourly outdoor time was higher during the day until afternoon. However, during the evening hours (5 PM to 8 PM), although hourly outdoor time decreased, hourly MIMS remained high.

Because sex was found to be a modifier in the association between daily outdoor time and daily total MIMS (Supplementary Fig. 1), we conducted a sex-specific multivariable regression analysis. Age-adjusted linear regression models showed that an additional 10 min of daily outdoor time was associated with an additional 271 daily total MIMS (95% CI = 110, 431) among boys and an additional 482 daily total MIMS (95% CI = 306, 659) among girls. When the models were further adjusted for confounding factors (Table 2), the coefficient remained statistically significant: an additional 10 min of daily outdoor time was associated with an additional 296 daily total MIMS (95% CI = 131, 460) among boys and an additional 540 daily total MIMS (95% CI = 372, 708) among girls. Sensitivity analysis for a subsample with ≥3 valid accelerometer wear days showed consistent results: an additional 10 min of daily outdoor time was associated with an additional 253 daily total MIMS (95% CI = 102, 406) among boys and an additional 446 daily total MIMS (95% CI = 282, 610) among girls. In addition, being a Hispanic ethnicity and a Black race were associated with higher daily total MIMS only among girls.

**Table 2:** Multivariable linear regression models to predict daily total MIMS and object control standard score in 2012 NNYFS participants aged 3 to 5 years

	Outcome: Daily total MIMS		Outcome: Object control standard score
	Boys (*n* = 152)	Girls (*n* = 149)	All (*n* = 291)
	Coefficient (95% CI)	Coefficient (95% CI)	Coefficient (95% CI)
**Intercept**	12,898 (9736, 16,059)	10,172 (7695, 12,648)	7.5 (6.8, 8.2)
**Age**			
4 vs. 3 years	2042 (− 422, 4507)	544 (− 1162, 2251)	NA^a^
5 vs. 3 years	3136 (690, 5583)	286 (− 822, 1393)	NA^a^
**Race/ethnicity**			
Hispanic vs. non-Hispanic White	201 (− 2043, 2445)	2955 (2096, 3814)	0.5 (−0.2, 1.2)
Non-Hispanic Black vs. non-Hispanic White	1341 (− 229, 2910)	1744 (129, 3420)	0.6 (− 0.1, 1.3)
Other vs. non-Hispanic White	−149 (− 2531, 2232)	1693 (− 261, 3647)	− 0.8 (−2.7, 1.1)
**Ratio of family income to poverty**			
< 1.0 (below the poverty line) vs. ≥3.0	1672 (− 229, 3572)	− 495 (− 2983, 1993)	−0.04 (− 0.9, 0.9)
1.0 to < 3.0 vs. ≥3.0	880 (− 1053, 2814)	1056 (− 1043, 3154)	− 0.2 (− 0.9, 0.4)
**Sibling(s) ≤ 5 years old in household**: yes vs. no	− 350 (− 1596, 897)	1187 (−606, 2980)	NA^b^
**Sibling(s) 6–17 years old in household**: yes vs. no	− 1146 (− 2251, −40)	−229 (− 2005, 1548)	0.6 (0.04, 1.2)
**Additional 10 min of daily outdoor time**	296 (131, 460)	540 (372, 708)	0.1 (0.001, 0.1)

*CI* confidence interval, *MIMS* monitor-independent movement summary

^a^Not applicable: the object control standard score was an age-adjusted score

^b^not applicable: living with child(ren) 6–17 years old was not included in the regression model, because it was statistically insignificant in bivariate analysis

In examining the association between outdoor time and gross motor competency outcomes, we found that sex was not a modifier. Sex-combined analysis showed that every additional 10 min of outdoor time was associated with a 0.1-point (95% CI = 0.001, 0.1) higher object control standard score (Table 2), but it was not associated with the gross motor quotient or the locomotor standard score (data not shown). Daily total MIMS was positively associated with the locomotor standard score, whereas it was not associated with the gross motor quotient score or the object control standard score (Supplementary Table 1). When we further compared gross motor competency outcomes by outdoor time and daily total MIMS, daily outdoor time remained significantly positively associated with the object control score (Table 3).

**Table 3:** Multivariable linear regression models to predict the gross motor outcomes by daily outdoor time and daily total MIMS in 2012 NNYFS participants aged 3 to 5 years (*n* = 291)

	Locomotor standard score	Object control standard score
	Coefficient (95% CI)	Coefficient (95% CI)
Intercept	7.4 (6.1, 8.8)	7.9 (6.6, 9.3)
Race/ethnicity		
Hispanic vs. White	0.6 (−0.4, 1.7)	0.5 (− 0.2, 1.2)
Black vs. White	1.5 (0.2, 2.8)	0.6 (−0.1, 1.3)
Other vs. White	−0.2 (−2.9, 2.5)	− 0.8 (− 2.7, 1.1)
Ratio of family income to poverty		
< 1.0 (below the poverty line) vs. ≥3.0	0.2 (−0.9, 1.4)	− 0.004 (− 1.0, 0.9)
1.0 to < 3.0 vs. ≥3.0	0.7 (− 0.6, 1.9)	− 0.2 (− 0.8, 0.4)
Sibling(s) 6–17 years old in household: yes vs. no	NA^a^	0.6 (0.2, 1.2)
Additional 10 min of daily outdoor time	0.01(−0.1, 0.1)	0.1 (0.01, 0.14)
Daily total MIMSx10^−3^	0.1 (−0.002, 0.2)	−0.03 (− 0.1, 0.1)

*CI* confidence interval, *MIMS* monitor-independent movement summary

^a^Not applicable: living with child(ren) 6–17 years old was not included in the regression model, because it was statistically insignificant in bivariate analysis

## Discussion

In a representative sample of U.S. preschool-aged children, we found that daily outdoor time was positively associated with daily PA, with the contribution of outdoor time to PA larger among girls than boys. Daily outdoor time was also statistically positively associated with object control competency; however, the effect size was marginal.

This study is one of the first to objectively quantify outdoor time throughout the day in a representative sample of U.S. preschool-aged children. We found that U.S. preschool-aged children spent on average 95 min/day outdoors. Earlier, Raustorp et al. [13] reported 92 min of observation-based outdoor time during on average 8 h of childcare attendance among 26 preschool-aged children in Raleigh, North Carolina. Tandon et al. [19] reported 74 min of GPS-measured outdoor time during on average 5 h of childcare attendance (24% of attendance time) among 46 preschool-aged children in metropolitan Seattle childcare centers. Although not directly comparable, given that the present study showed 77 min of outdoor time during a 8-h period (between 9 AM and 5 PM; Fig. 1) or 50 min of outdoor time during a 5-h period with more outdoor time (between 11 AM and 4 PM; Fig. 1), our U.S. national estimate of outdoor time in a representative sample appears to be lower, compared to the sample in the Raustorp study [13] or the Tandon study [19]. We also found that outdoor time did not differ between boys and girls. This finding is inconsistent with prior studies [10, 19, 27, 28] that reported lower outdoor time among girls than boys in early childhood. The potential sex difference in outdoor time should be further examined with an objective measure, considering various correlates of outdoor time [28]. In addition, the characteristics of the outdoor spaces should be considered, since outdoor features, such as open areas, pathways, and nature elements, can influence health-related behaviors [7, 29].

The present study used daily total MIMS as an accelerometer-based PA metric. MIMS is a newly developed universal summery metric that accounts for discrepancies in raw data across accelerometer devices to overcome the limitation of incomparable device-specific accelerometer counts [23]. The publicly available NHANES 2011–2014 accelerometer datasets contain MIMS metric data, without other traditional accelerometer metrics, such as ActiGraph-specific accelerometer counts. Although a universal metric, such as MIMS, is not yet widely utilized, the use of MIMS in a national survey will encourage researchers to consider using this universal metric, so as to produce comparable PA data across studies moving forward. A recent publication of the R package, “MIMSunit” [30], is expected to help researchers easily compute MIMS units and, therefore, accelerate the advancement of research practice. Future studies should further validate MIMS.

Prior studies [3–7] have reported that in the preschool years, girls were less active than boys. In contrast, the present study found that daily PA levels were similar between boys and girls in a representative sample of U.S. preschool-aged children. Furthermore, the present study found that the contribution of outdoor time to PA was larger among girls than boys, which is inconsistent with the finding in the Tandon study [19] that reported a similar level of contribution between boys and girls but was based only in childcare settings. As illustrated in Supplementary Fig. 1, with zero outdoor time, boys tended to be more active than girls. However, as daily outdoor time reached approximately 110 min, daily total PA level was the same between boys and girls. Moreover, with further increases in daily outdoor time, girls engaged in more PA than boys. Conversely, the negative association between daily indoor time and daily PA was stronger among girls than boys (data not shown). Our findings suggest that external support such as taking preschool-aged children outside is important for PA promotion, particularly among girls.

In the U.S., two in three preschool-aged children attend childcare [31]. Because of its broad reach, childcare is considered an important setting for PA promotion in young children. Based on the scientific evidence, including clinical trials [32, 33], current National Health and Safety Performance childcare standards [34] recommend providing outdoor active play time year-round two or more times per day totaling 60 to 90 min. Although the present study did not specifically examine outdoor time and PA in a childcare setting, the findings of the study support the importance of such outdoor childcare policies to promote equitable PA at a population level. In practice, however, several barriers exist to following these recommendations, including weather conditions, limited outdoor space within a childcare property, suboptimal child outerwear/shoes, and concerns about sun exposure [7, 35, 36]. To overcome these barriers, several strategies have been suggested, such as scheduling multiple shorter periods of outdoor time [32] and utilizing publicly available outdoor spaces (e.g., parks and playgrounds) [7]. Physical and social safety of young children should also be balanced with opportunities for child development through outdoor activities [37]. Collaboration with local crime prevention and other community-based organizations and programs (e.g., adaptation of the “Safe Routes to Schools” model [38]) can help mitigate safety concerns regarding the use of community outdoor spaces.

The present study found no association of light sensor-measured outdoor time with total motor skill or locomotor skill competency, but a positive association with object control competency among U.S. preschool-aged children. However, an additional 10 min of daily outdoor time was associated with only a 0.1-point higher object control score, which is of questionable clinical significance. A review in 2014 [39] found very low-quality evidence regarding the association between outdoor time and motor skills, identifying only one study that reported no association between outdoor time and motor skill competency among preschool-aged children. Since then, Barnett et al. [11] showed that self-reported outdoor time at age 3.5 years was not prospectively associated with object control or locomotor competency at age 5 years among 178 Melbourne Infant Feeding, Activity, and Nutrition Trial participants. Niemistö et al. [10] showed statistically positive correlations between self-reported outdoor time and both object control and locomotor competencies in a representative sample of 945 Finnish preschool-aged children; however, the strength of the correlation was low (*r* < 0.13). It is plausible that the contribution of outdoor time to object control competency is partly mediated though higher PA. However, the present study did not find a significant association between PA and object control competency, consistent with previous findings [26, 40]. The lack of association could be partly because the 60-s epoch MIMS metric is unable to fully capture specific movements without locomotion (e.g., kick, catch) that can contribute to the development of object control skills [40]. Qualitative information about activity types, for example, machine learning-based pattern recognition, may provide greater insights into the relationship between PA and object control competency. The contribution of outdoor time to object control competency could also be explained by the characteristics of the outdoor play environment. For example, we speculate that it would be easier to work on ball skills in a more open outside environment and parents are likely more amenable to their preschool-aged children using balls outdoors than inside. The mechanisms underlying the observed weak association between outdoor time and object control competency should be further investigated.

A few limitations of this study should be acknowledged. First, outdoor darkness would have lowered the light sensor-based indoor/outdoor detection accuracy, which might have caused an underestimation of outdoor time during evening hours. However, it is not common for preschool-aged children to be outside when it is dark. Second, because the MIMS approach is a fairly new data processing method, our findings may not be directly comparable with the findings of previous studies that did not use the MIMS approach. Third, data from 2012 could differ from data collected today. Lastly, we were unable to account for potentially important confounding factors, such as seasonality, weather, outdoor brightness/darkness, childcare attendance, and parental influences, as those data were unavailable.

## Conclusions

In a representative sample of U.S. preschool-aged children, daily outdoor time was positively associated with daily PA. The contribution of outdoor time to PA was larger among girls than boys, suggesting that providing opportunities for preschool-aged children to be outdoors is critical for promoting PA, particularly among girls.

## Supplementary Information

**Additional file 1: Supplementary Fig. 1.** Scatter plots for daily outdoor time and daily total MIMS in 2012 NNYFS participants aged 3 to 5 years (*n* = 301). **Supplementary Table 1.** Multivariable linear regression models to predict gross motor outcomes by daily total MIMS in 2012 NNYFS participants aged 3 to 5 years (*n* = 291).

## Data Availability

The data that support the findings of this study are available from https://www.cdc.gov/nchs/nnyfs/index.htm.

## Abbreviations

CI: Confidence interval
GPS: Global Positioning System
IQR: Interquartile range
MIMS: Monitor-independent movement summary
NHANES: National Health and Examination Survey
NNYFS: National Health and Examination Survey National Youth Fitness Survey
PA: Physical activity
PAM: Physical activity monitor
TGMD-2: Test of Gross Motor Development-Second Edition
