# Supplementary material for: Cross-sectional association of light sensor-measured time outdoors with physical activity and gross motor competency among U.S. preschool-aged children: the 2012 NHANES National Youth Fitness Survey
Source: BMC Public Health. 2022 Apr 26;22:833. doi: 10.1186/s12889-022-13239-0 (PMC9040315; doi:10.1186/s12889-022-13239-0)
Supplement: Supplementary file 1 — Additional file 1: Supplementary Fig. 1. Scatter plots for daily outdoor time and daily total MIMS in 2012 NNYFS participants aged 3 to 5 years (n = 301). Supplementary Table 1. Multivariable linear regression models to predict gross motor outcomes by daily total MIMS in 2012 NNYFS participants aged 3 to 5 years (n = 291). [file 12889_2022_13239_MOESM1_ESM.docx]

**Supplementary Data**

Supplementary Figure 1. Scatter plots for daily outdoor time and daily total MIMS in 2012 NNYFS participants aged 3 to 5 years (n=301).


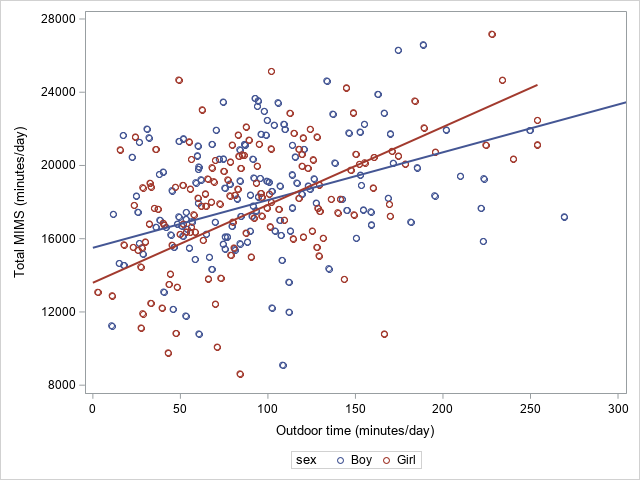


MIMS, monitor-independent movement summary

Supplementary Table 1. Multivariable linear regression models to predict gross motor outcomes by daily total MIMS in 2012 NNYFS participants aged 3 to 5 years (n=291).

|  | Gross motor quotient | Locomotor  standard score | Object control  standard score |
| --- | --- | --- | --- |
|  | Coefficient (95% CI) | Coefficient (95% CI) | Coefficient (95% CI) |
| Intercept | 86.4 (79.1, 93.6) | 7.4 (6.1, 8.8) | 7.9 (6.5, 9.3) |
| Race/ethnicity |  |  |  |
| Hispanic vs. White | 3.1 (-1.7, 7.9) | 0.6 (-0.5, 1.7) | 0.4 (-0.3, 1.0) |
| Black vs. White | 5.9 (1.0, 10.9) | 1.5 (0.2, 2.7) | 0.5 (-0.1, 1.1) |
| Other vs. White | -3.3 (-17.2, 10.5) | -0.2 (-2.9, 2.5) | -0.9 (-2.8, 0.9) |
| Ratio of family income to poverty |  |  |  |
| <1.0 (below the poverty line) vs. ≥3.0 | -0.1 (-5.3, 5.4) | 0.2 (-0.9, 1.4) | -0.1 (-1.0, 0.8) |
| 1.0 to <3.0 vs. ≥3.0 | 1.0 (-4.4, 6.3) | 0.7 (-0.6, 1.9) | -0.3 (-1.0, 0.4) |
| Sibling(s) 6-17 years old in household: yes vs. no | 2.3 (-1.0, 5.6) | NA^a^ | 0.7 (0.1, 1.3) |
| Daily total MIMSx10^-3^ | 0.4 (-0.1, 0.8) | 0.1 (0.02, 0.2) | 0.02 (-0.07, 0.1) |

CI, confidence interval; MIMS, monitor-independent movement summary.

^a^Not applicable: living with child(ren) 6-17 years old was not included in the regression model, because it was statistically insignificant in bivariate analysis.
